# Laser Micromachining of Lithium Niobate-Based Resonant Sensors towards Medical Devices Applications

**DOI:** 10.3390/s20082206

**Published:** 2020-04-14

**Authors:** Zeyad Yousif Abdoon Al-Shibaany, Pavel Penchev, John Hedley, Stefan Dimov

**Affiliations:** 1 Biomedical Engineering Department, University of Technology-Iraq, Baghdad 10066, Iraq; 2 Cardiff School of Engineering, Cardiff University, Cardiff CF24 3AA, UK; 3 Department of Mechanical Engineering, The University of Birmingham, Birmingham B15 2TT, UK; P.Penchev@bham.ac.uk (P.P.); S.S.Dimov@bham.ac.uk (S.D.); 4 School of Engineering, Newcastle University, Newcastle upon Tyne NE1 7RU, UK; john.hedley@ncl.ac.uk

**Keywords:** laser micromachining, lithium niobate, sensors, biosensors, medical devices

## Abstract

This paper presents a micromachining process for lithium niobate (LiNbO_3_) material for the rapid prototyping of a resonant sensor design for medical devices applications. Laser micromachining was used to fabricate samples of lithium niobate material. A qualitative visual check of the surface was performed using scanning electron microscopy. The surface roughness was quantitatively investigated using an optical surface profiler. A surface roughness of 0.526 μm was achieved by laser micromachining. The performance of the laser-micromachined sensor has been examined in different working environments and different modes of operation. The sensor exhibits a Quality-factor (Q-factor) of 646 in a vacuum; and a Q-factor of 222 in air. The good match between the modelling and experimental results shows that the laser-micromachined sensor has a high potential to be used as a resonance biosensor.

## 1. Introduction

Transparent materials, such as lithium niobate, can be micromachined by Femtosecond laser micromachining technique [1]. Due to its pre-eminent optical, electronics, and physical properties, lithium niobate has a wide range of applications, such as optical devices, surface acoustic wave sensors, intensity modulator, and radio telecommunications [2,3]. Additionally, lithium niobate material is used widely in micro-electromechanical system MEMS applications due to its superior piezoelectric properties. The common microfabrication processes require a significant capital investment due to the use of clean room facilities. The ability to produce MEMS-based sensors using new micromachining techniques offers a low-cost and sustainable technology, provided production volumes are low. The high overhead costs associated with classical microfabrication results in the prototyping of designs typically costing in the range of £10,000 to £100,000 and with process flows not yet optimised for such designs. The use of CNC micromachining [4,5] and laser micromachining has a significant impact on the production cost of sensors where the cost of fabrication can be reduced significantly. 

Biosensing may include the sensing of biochemicals, molecules, proteins, or any other structure to determine as a cell. An ultrahigh sensitivity (high-quality factor) and lower detection limit are required in biosensing applications. Lithium niobate has been used to fabricate sensors for biosensing applications. In 1993, Zhang et al. from the National Microelectronics Research Centre in Ireland reported two acoustic wave transducers that were optimised for biosensing applications in a liquid medium, and both transducers employ the lithium niobate piezoelectric characteristics [6]. Nanzin and Sher reported a design of a lithium niobate-based optical mirroring resonator for biosensing applications where the sensors offer a sensitivity of approximately 68 nm/refractive index unit (RIU) and a minimum detection limit of 10^−2^ RIU [7]. Suarez et al. showed that proteins could be bound onto lithium niobate waveguides, which is the first step towards the development of integrated biosensors [7]. For resonant-based biosensing, it is required to have a good quality (ideally smooth) surface for immobilisation, a high-quality factor for the accurate measurement of the frequency, whilst having a cost-effective production method for what needs to be a disposable technology. It is also advantageous to have a cost-effective prototyping manufacture to facilitate the technology on its route to commercialisation.

In this work, the micromachining of lithium niobate for the rapid prototyping of a circular diaphragm resonant biosensor is assessed using laser micromachining. The process is optimised to provide high-quality, economic, and sustainably manufactured lithium niobate sensor prototypes for biosensing. The application of micromachining is expected to open new industrial avenues for prototyping biosensors. The proposed sensor is first being modelled by ANSYS to find the natural frequencies and to choose some modes of operation so that the manufactured sensor can be assessed later in terms of frequencies and Q-factors. A surface roughness of 0.526 μm was achieved by the laser micromachining. The performance of the laser-micromachined sensor has been examined in vacuum and air, and in different modes of operation. The natural frequencies of the sensors are experimentally measured by two different techniques: by using the vibrometer and by electrical measurements. The good match between the modelling and experimental results shows that the laser-micromachined sensor has a high potential to be used as a resonance biosensor. A Quality-factor (Q-factor) of 646 is achieved in a vacuum, and one of 222 is achieved in air. In addition, the use of chemicals and the chemical wastes resulting from the use of cleanroom facilities have been eliminated from the manufacturing process, which results in a more sustainable and environmentally friendly manufacturing process.

The rest of the paper is organised as follows: Section 2 explains the methodology that has been followed during the work and the experimental setup of all the equipment of the experiments. Section 3 describes the optimisation of laser parameters for the micromachining process. The results of the micromachining process are discussed in Section 4. Section 5 shows the performance of the sensor. Finally, the paper ends with the conclusions in Section 6.

## 2. Methodology and Experimental Setup

### 2.1. Laser Platform Setup

The micromachining of any transparent material, such as lithium niobate, using a laser is not an easy process due to the difficulty of energy transfer mediation from the light to the material by linear absorption [8]. Femtosecond laser processing is a photochemical process, characterised by ultrashort pulse durations (ps and fs) that are shorter than the material thermalisation time. The photochemical laser process is characterised by non-linear absorption phenomena due to the extremely high energy intensities of the ultrashort laser pulses, and the material removal mechanism is called multiphoton absorption. In multiphoton absorption, the bound electrons of the material can be directly freed from the valence band by absorbing multiple photons. The direct ionisation and the formation of dense electron-hole plasma lead to thermal material transformations: bond breaking and the explosive disintegration of the lattice through electron repulsion, which makes it possible to process any material including transparent substrates such as glass or LiNbO_3_, as is the case with the reported research in this paper. Laser microfabrication techniques have been used to fabricate lab-on-chip and MEMS [9,10]. Laser micromachining is also used to develop micro-mixers and micro-pumps to fabricate lab-on-chip devices [11,12,13]. In this work, laser micromachining has been utilised as an alternative sustainable manufacturing technique to fabricate lithium niobate-based sensors.

**Figure 1:**
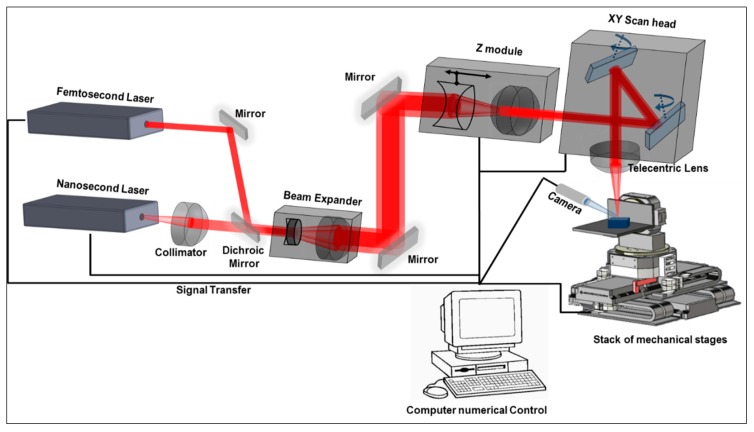
Schematic representation of the laser microprocessing platform.

Experimental tests were performed on a state-of-the-art laser micromachining (LMM) platform, which incorporates three linear stages and two rotary stages, and a 3D scan head (three optical axes) to move the beam spot with high dynamics within a 35 mm × 35 mm × 6 mm processing envelope. The positioning resolutions of the linear and rotary stages, as stated by the manufacturer, are 0.25 µm and 45 µrad, respectively [14]. The accuracy, repeatability, and reproducibility (ARR) achievable with the 3D scan head is better than +/− 5 µm across the full range of scanning speeds [15]. The LMM platform integrates a Yb-doped femtoseconds (fs) 5W laser source from Amplitude Systems that operates at a central wavelength of 1030 nm and has a maximum repetition rate of 500 KHz. Furthermore, the LMM platform is equipped with a 100-mm telecentric focusing lens that provides a beam spot diameter of 30 µm at the focal plane and a machining volume with the scanner of 35 mm (X) × 35 mm (Y) × 6 mm (Z). The fs laser source is employed in the experiments in order to minimise the heat-affected zone around the processed area and to thus reduce the thermally induced cracks and damages around the processed area. At the same time, the optical axes are employed to realise the required laser beam movements on the workpiece; this is done in order to benefit from the high processing speeds achievable with the 3D scan head (up to 2 m/s), and thus to obtain high machining throughputs. The laser microprocessing platform employed in this research is schematically shown in Figure 1.

### 2.2. Vibrometer and Chamber Setup

**Figure 2:**
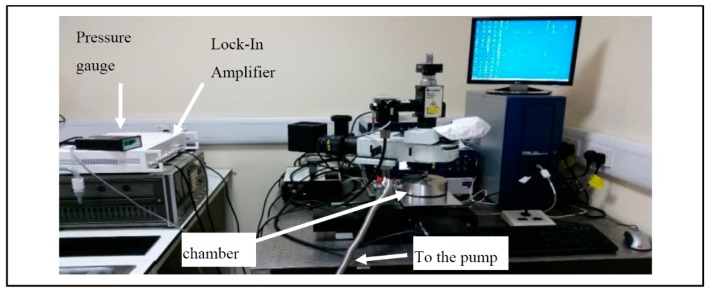
Vibrometer test station arrangement.

The physical arrangement is shown in Figure 2.

The vibrometer test station was used to characterise the sensor and test the functionality of the novel electrode design. The sensor mode shapes were mapped using the Polytec UHF 120 vibrometer system. The sensor was placed inside a vacuum chamber to allow for testing under vacuum conditions, as well as under atmospheric pressure. The Polytec OFV-501 fibre optic interferometer and Polytec OFV-3000 controller unit were used in this test. The vibrometer system utilised a single-point laser beam [16] that was aligned and directed perpendicularly onto the vibrating structure. This testing station measured only the out-of-plane vibration.

**Figure 3:**
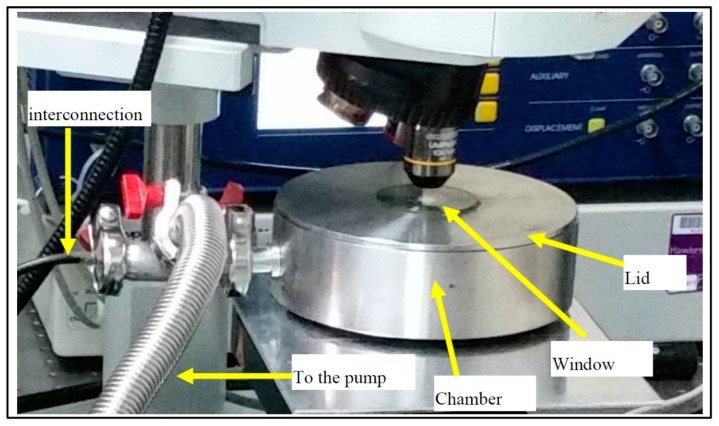
Test chamber.

The test station had a programmable lock-in amplifier (Model HF2LI) (Zurich Instruments), which was used to drive the sensor with a precise voltage signal. The same lock-in amplifier can be used for both sensing and actuating the sensor; however, in this case, it was used to send out the actuating signal, while the sensing was performed by the vibrometer. To measure the resonance frequency of the sensor, an AC voltage signal can be sent with a frequency range of up to 50 MHz and a maximum amplitude of 5 V. In this experiment, the sensor was actuated by a 1 V signal and a frequency of up to 2 MHz, which covered the first 200 modes of operation of the sensor. The range of the frequency of the vibrometer is up to 20 MHz with a sensitivity of 50 nm/V. To measure the sensor vibration, a beam from a single-point laser was used. The test station had the ability to map the mode shape of the sensor vibration by scanning the full sensor using the single-point laser beam. The sensor was place on the X–Y station in order to allow for full sensor mapping. The motorised X–Y stage was controlled by the computer, which offered a precise movement with ±1 μm accuracy. Both the actuation signal and the laser-detected signal from the vibrometer were digitised, and the amplitude and phase signals were computed as a function of the frequency. The results could then be read directly on the user control panel and were easily downloaded as an Excel spreadsheet for offline use. The user interface offers the ability to control the amplitude of the driving signal, the frequency range, and the number of data points, as well as other controls to provide robust output data. The testing chamber in Figure 3 was used in the characterisation process. The chamber was used to control the pressure under which the sensor was tested. The minimum pressure achieved in this chamber was 7 mbar. 

The lid of the chamber had a glass window to allow the laser to scan the sample. When the air was pumped out of the chamber, the lid was pushed down by the atmospheric pressure to fully compress a rubber washer located between the lid and the chamber. The interconnection (feedthrough) line was fully sealed with epoxy. The pressure was measured and monitored using a pressure gauge.

### 2.3. Microscope

**Figure 4:**
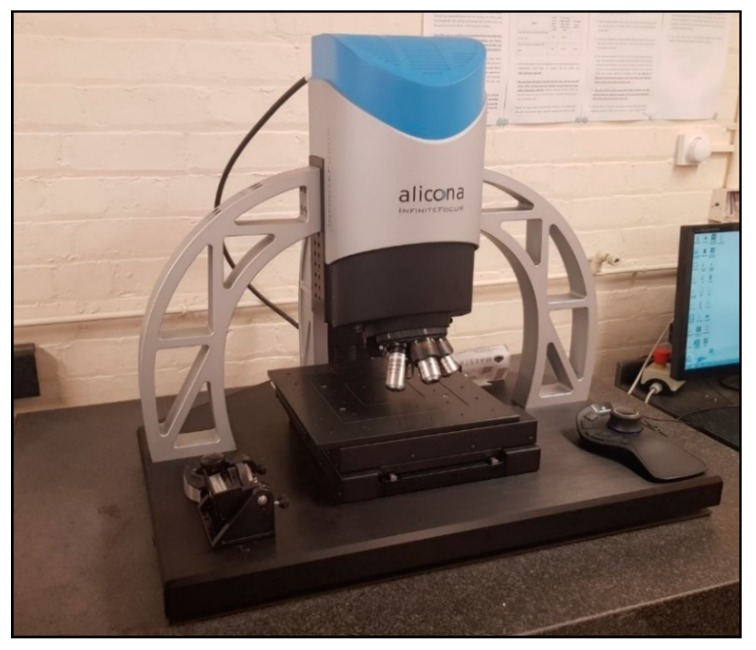
The Focus Variation 3D microscope, Alicona Infinite Focus G5.

Measurements and analyses of laser-processed areas were performed with an optical Focus Variation (FV) 3D microscope, namely Alicona Infinite Focus G5, shown in Figure 4 below. It has ×5, ×10, ×20, ×50 objective, and x100 lenses that provide lateral resolutions of 3.52 µm, 1.76 µm, 0.88 µm, 0.64 µm, and 0.44 µm and vertical resolutions of 0.41 µm, 0.1 µm, 0.05 µm, 0.02 µm, and 0.01 µm, respectively. In this research, the ×10 objective lens was employed to measure the laser-processed areas.

## 3. Optimisation of Laser Parameters

The laser processing parameters were optimised for micromachining lithium niobate sensors, where the goal was to obtain a surface roughness that was as low as possible while the material removal rates should be as high as possible in order to reduce the laser processing times. The following laser parameters were considered in the conducted optimisation trials: laser pulse energy and laser machining strategy (pulse distance, hatch pitch, and hatch angle between laser machining layers). The ranges of processing parameters for the optimisation trial were selected to encompass the complete technical capabilities of the fs laser source. Table 1 below summarises the different levels of parameter settings that were used.

**Figure 5:**
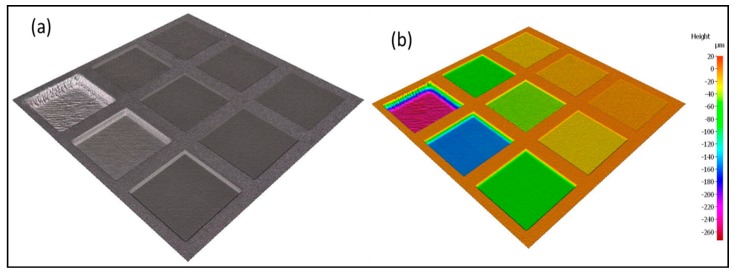
(**a**) 3D view of the nine machined 2 mm × 2 mm squares during the laser parameter optimisation trials; (**b**) pseudo-colour representation of (**a**).

The machining geometry for the optimisation trials was conducted on a 2 mm × 2 mm square substrate, as shown in Figure 5a,b below, where each square pocket was produced with ten machining layers along the z axis.

**Figure 6:**
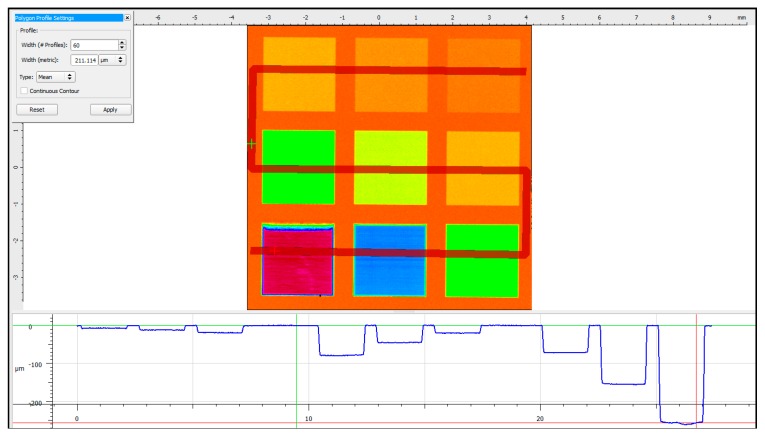
Surface profile of the optimisation samples.

The results from the surface profile measurements conducted using the FV microscope on all nine laser-machined square pockets are shown in Figure 6.

**Figure 7:**
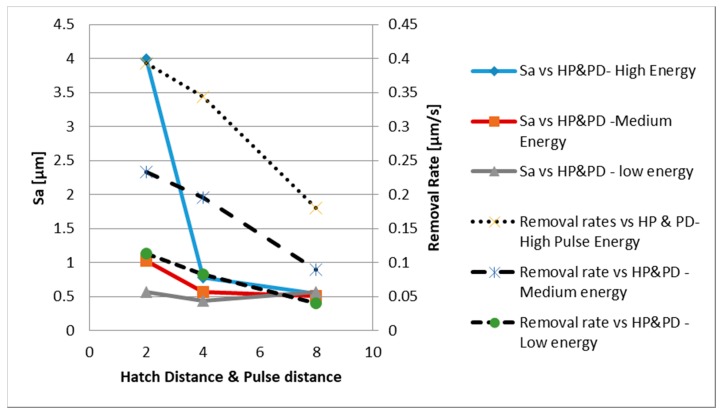
Optimisation of laser parameters to obtain a low Sa (average surface roughness) and high removal rates.

Figure 7 depicts a plot of the significant dependences that were observed between the machining results and the laser parameter settings with regard to the material removal rates and surface integrity.

The results from the optimisation trials as depicted in Figure 7 can be summarised as follows:

- The highest removal rates (0.39 µm/s) were obtained with the smallest hatch pitch and pulse distance (2 µm) and the highest pulse energy (7.8 µJ).
- The effect of the hatch pitch and pulse distance on the resulting Sa was very much dependent on the used laser energy; in particular, the hatch pitch and pulse distance had a high impact on Sa at the highest pulse energy, while they had a negligible effect at the lowest energy setting.
- The lowest Sa (Sa = 0.431 µm) was obtained with a hatch pitch and pulse distance of 4 µm and the lowest pulse energy (3 µJ).

Based on the performed experimental trials, it could be stated that the best combination of parameters is a high pulse energy (7.8 µJ) and hatch pitch and a pulse distance of 4 µm. Table 2 also summarises all the other laser parameter settings which have been used for the production runs.

## 4. Micromachining Results 

**Figure 8:**
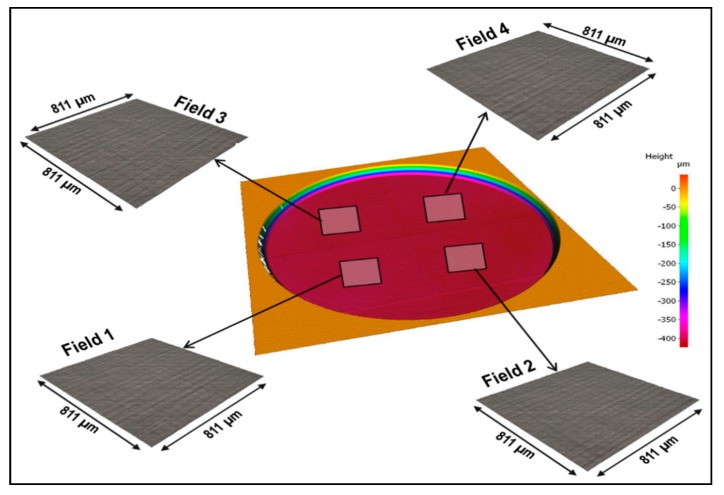
Laser micromachining results obtained with the optimised laser parameter settings to fabricate a 7-mm diameter circular recess with a depth of 400 µm.

A 7-mm diameter circular recess with a depth of 400 µm that was laser-machined with the optimised laser parameter settings from Table 2 is depicted in Figure 8. In addition, it shows four fields that were scanned at the bottom surface of the laser-machined recess in order to evaluate the resulting surface roughness. 

The surface quality of the micromachined sample in Figure 8 was considered of sufficiently good quality, especially with no cracks on the main surface of the membrane. There were some debris left on the surface, but they were removed easily through ultrasonic cleaning with water to ensure that the surfaces of the fields that were used to analyse the resulting roughness were free from any debris. The obtained area-based surface roughness parameters of the four fields are provided in Figure 8 and Table 3.

For mass sensors, average roughness values ranging from 0.149 µm to 0.75 µm were reported in [17]. Furthermore, it was reported in the same work that for resonators with a large thickness (e.g., 50 µm), the natural frequencies are unchangeable due to a surface roughness variance of 1 µm. For the sensor in this work, the thickness is 100 µm, and the roughness (with no post-processing) ranges from 0.526 µm to 0.545 µm.

## 5. Sensor Performance

### 5.1. Finite Element Analysis (FEA) of the Sensor by ANSYS

The finite element (FE) model of the sensor was modelled in ANSYS for simulation purposes. ANSYS Parametric Design Language (APDL) was used as the programming language in this work in order to allow for batch processing rather than using the graphical user interface (GUI). To simulate the piezoelectric material, it is necessary to couple the electrical and structural fields in the same simulated material. The sensor was modelled in ANSYS as a 3D 20-node coupled-field solid called SOLID226. SOLID226 is piezoresistive, electroelastic, piezoelectric, and has many other properties. The SOLID226 element has 20 nodes, with up to five degrees of freedom per node. Structural capabilities include elasticity, plasticity, viscoelasticity, large strain, large deflection, stress stiffening effects, and pre-stress effects [18]. SOLID226 has been used previously by many researchers to simulate piezoelectric material as well as for the design optimisation of piezoelectric material [19], where high-precision solutions can be obtained using this element type. The material properties of the lithium niobate were input as matrices. ANSYS uses a convention that is different from the widely used method [20] for matrix ordering. The necessary corrections were made to conform to the ANSYS conventions. The properties of lithium niobate are reported in several studies [2,21], and the most cited summary of lithium niobate material properties is reported in [22]. The critical part of any finite element analysis is the setting of boundary conditions. The model of the sensor is a circular plate clamped at the edge, which means that there is no displacement at the edge of the design. All the nodes at the circle’s circumference were fixed in all directions so that there was no displacement in the x, y, or z directions. However, the rest of the boundaries were free.

### 5.2. Vibrometer Measurement

All modes of operation were measured with a vibrometer. The advantages of vibrometer measurement are that it has an easy measurement setup and a very good signal-to-noise measurement. This technique was feasible for the measurement of the out-of-plane modes. However, in-plane mode measurement is problematic because the laser can only detect out-of-plane displacement.

**Figure 9:**
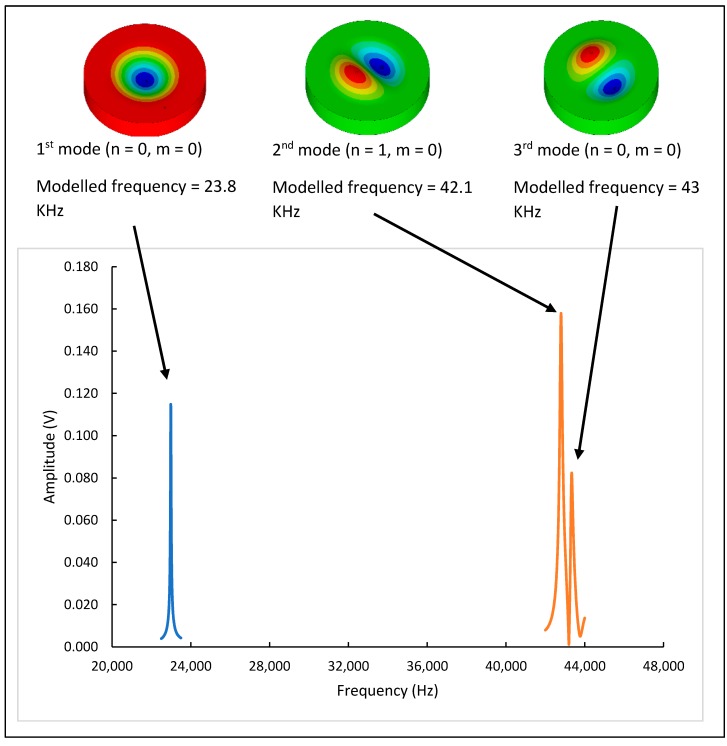
First three mode shapes with corresponding measured frequencies.

The sensor was actuated with a sine sweep with a 1 V peak voltage and a frequency range of up to 1.5 MHz to cover the first 200 modes. Figure 9 shows the plot of the first three modes and the corresponding measured frequencies. The results obtained in the vibrometer experimental measurement are in agreement with those obtained using ANSYS.

### 5.3. Mode Mapping

**Figure 10:**
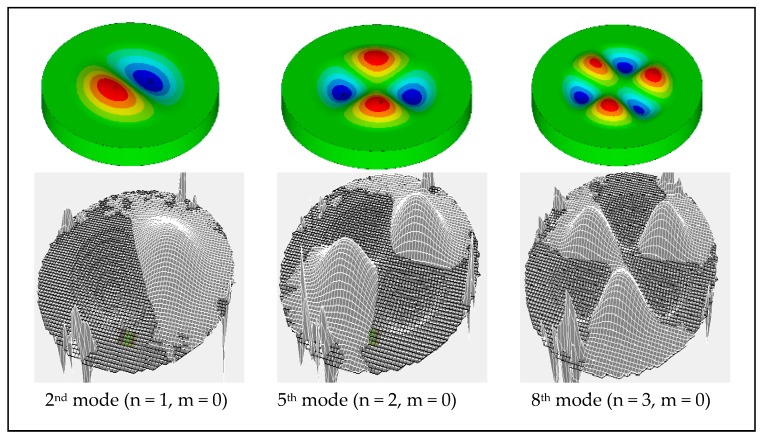
Examples of the experimental mode mapping.

Another experimental measurement was conducted to map the mode shapes of the actual sensor using the vibrometer. The results obtained from the test, as shown in Figure 10, prove that the ANSYS model of the sensor was the best representation.

**Figure 11:**
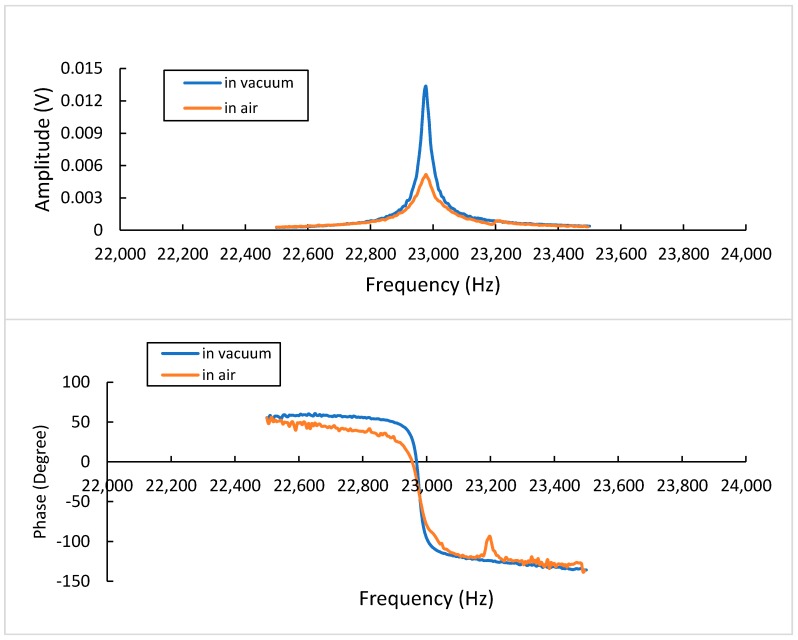
Frequency response of mode (0,0)-vibrometer measurement.

The mapping of the mode shape results in Figure 11 shows some spikes that represent random data. However, these spikes do not affect the results, as they are located outside the sensor area; the reason for having them is that the area outside the sensor is not fully covered with gold, so the laser is not reflected here because lithium niobate is a transparent material. The mapping measurements were conducted to cover the first 200 modes; however, not all of them were mapped because the electrode design preferentially drives/detects particular modes. The out-of-plane modes predominantly show up in the mapping process.

### 5.4. Experimental Verification 

This section will discuss how the sensor was examined in different working environments and different modes of operation. As mentioned previously, the performance evaluation was conducted using two measurement techniques: the vibrometer and electrical measurements. The first mode to be investigated was mode (0,0), shown in Table 4. ANSYS reported that the frequency of mode (0,0) was 23.85 kHz. The vibrometer measurement of the frequency response of this mode, which was conducted both in a vacuum and air, is shown in Figure 11. The frequency of mode (0,0) was 22.9 kHz, as reported by the vibrometer measurement. The amplitude was damped by about 60% in air compared to the amplitude in a vacuum. The Q-factor of the sensor when working in mode (0,0) was 646 in a vacuum. However, this was significantly reduced in air (Q = 49).

The other mode of interest was mode (1,0), which had two associated frequencies, as reported by ANSYS. The mapping results shows that two modes appear, the mode shapes are identical but rotated by 90° to each other. With the amplitude responses overlapping at these two resonant frequencies, it is difficult to map each mode shape separately. The first frequency was 42.70 kHz and the second one was 43.10 kHz, and both have Q-factors of 366 in vacuum and 222 in air. The sensor was tested in two different environments, vacuum and air, in order to assess the quality factor and check the feasibility of using it for biosensing purposes. The sensor shows promising results for a potential resonance biosensor.

## 6. Conclusions

This work reported the micromachining of lithium niobate for the prototyping of a circular diaphragm resonant biosensor using laser micromachining. The micromachining process was optimised to provide high-quality, economical, and sustainably manufactured lithium niobate sensor prototypes. The proposed sensor was first modelled by ANSYS to find the natural frequencies and modes of operation that were chosen, and the sensor was assessed in terms of the working frequencies and Q-factors. A surface roughness of 0.526 μm was achieved by the laser micromachining. The performance of the sensor was examined in vacuum and air, and in different modes of operation. The natural frequencies of the sensors were experimentally measured by two different techniques: by using the vibrometer and by electrical measurement. A Quality-factor (Q-factor) of 646 was achieved in a vacuum, and one of 222 was achieved in air, and the good match between the modelling and experimental results showed that the laser-micromachined sensor has a high potential to be used for resonant-based biosensing as the sensor had a good-quality surface for and a high-quality factor for the accurate measurement of the frequency, whilst having a cost-effective production method for what needs to be a disposable technology. It is also advantageous to have cost-effective prototyping manufacture to facilitate the technology on its route to commercialisation.

## Figures and Tables

**Table 1:** Different levels of parameter settings.

Laser Parameter	Units	Level 1	Level 2	Level 3
Pulse energy	µJ	7.8 (maximum energy)	5 (medium energy)	3 (just above ablation threshold)
Pulse distance and hatch distance	µm	2	4	8

**Table 2:** Optimised laser parameters for the laser machining trials.

Laser Parameter	Units	Value
Power	W	3.9
Frequency	kHz	500
Pulse energy	µJ	7.8
Scanning speed	m/s	2
Pulse duration	fs	310
Beam diameter	µm	30
Laser beam polarisation	-	Circular
Hatch style	-	Random
Hatch pitch	µm	4
Layer thickness	µm	7.5
Machining rate	mm^3^/s	0.05

**Table 3:** Area-based surface roughness parameters of the four fields from Figure 8.

Surface Roughness Parameters	Field 1	Field 2	Field 3	Field 4
Sa [µm]	0.526	0.536	0.545	0.533
Sq [µm]	0.656	0.672	0.681	0.670
Sz [µm]	5.429	5.421	5.288	5.618
S10z [µm]	5.003	5.037	4.849	5.016
Sp [µm]	2.886	2.985	2.627	2.98
Sv [µm]	2.543	2.436	2.661	2.638

**Table 4:** First three modes (modes of interest).

Mode Number	Modelled Frequency (KHz)	Vibrometer Frequency (KHz) Vacuum	Electrical Measurement (KHz) Vacuum	Q-Factor in Vacuum	Q-Factor in Air
1	23.8	22.98	21.40	646	49
2	42.1	42.70	42.79	366	222
3	43.0	43.10	43.32	366	222
